# Microstructural Characterization of PCL-HA Bone Scaffolds Based on Nonsolvent-Induced Phase Separation

**DOI:** 10.1021/acsomega.3c05616

**Published:** 2023-12-06

**Authors:** Mehmet Serhat Aydin, Mervenaz Sahin, Zeynep Dogan, Gullu Kiziltas

**Affiliations:** † Department of Material Science and Nanoengineering, Faculty of Engineering and Natural Sciences, Sabanci University, Istanbul 34956, Turkey; ‡ Center for Translational Oral Research (TOR), Department of Clinical Dentistry, Faculty of Medicine, University of Bergen, Bergen 5009, Norway; § Department of Molecular Biology, Genetics and Bioengineering, Faculty of Engineering and Natural Sciences, Sabancı University, Istanbul 34956, Turkey; ∥ Department of Mechatronics, Faculty of Engineering and Natural Sciences, Sabanci University, Istanbul 34956, Turkey; ⊥ Sabanci University Nanotechnology Research and Application Center, Istanbul 34956, Turkey

## Abstract

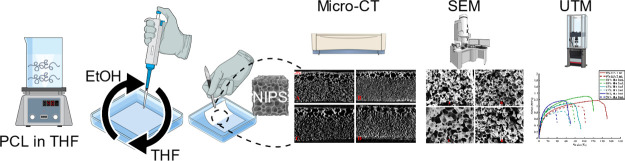

Composite materials containing pores play a crucial role in the field of bone tissue engineering. The nonsolvent-induced phase separation (NIPS) technique, commonly used for manufacturing membranes, has proven to be an effective method for fabricating composite scaffolds with tunable porosity. To explore this potential, we produced 10% (w/v) poly(caprolactone) (PCL)-nanohydroxyapatite (HA) composite porous film scaffolds with varying HA contents (0/10/15/20 wt %) and two thicknesses (corresponding to 1 and 2 mL of solution resulting in 800–900 and 1600–1800 μm thickness, respectively) using the NIPS method. We conducted a comprehensive analysis of how the internal microstructure and surface characteristics of these scaffolds varied based on their composition and thickness. In particular, for each scaffold, we analyzed overall porosity, pore size distribution, pore shape, and degree of anisotropy as well as mechanical behaviors. Micro-CT and SEM analyses revealed that PCL-HA scaffolds with various HA contents possessed micro (<100 μm) scale porosity due to the NIPS method. Greater thicknesses typically resulted in larger average pore sizes and greater overall porosity. However, unlike in thinner scaffolds, greater/higher HA content did not exhibit a direct correlation with a greater pore size for thicker scaffolds. In thinner scaffolds, adding HA above an effective threshold content of 15 wt % and beyond did lead to a greater pore size. The higher pore anisotropy was in line with the higher HA content for both groups. SEM images demonstrated that both groups showed highly uniformly distributed internal microporous morphology regardless of HA content and thickness. The results suggest that NIPS-based scaffolds hold promise for bone tissue engineering but that the optimal HA content and thickness should be carefully considered based on desired porosity and application.

## 1 Introduction

Bone fracture, one of the most widespread injuries, is associated with individual disability and loss of social productivity, resulting in very high treatment costs.^1^ Well-designed and engineered scaffold implants have proven to be an effective method for promoting successful healing. To achieve this, 3D composite porous tissue scaffolds in combination with bioactive molecules and cells have gained attention for the repair of damaged tissues. It is well-known that bone repair is a complex process, and when bone scaffolds are employed, success has been closely linked to specific attributes, including (i) mechanical support for the growth and functioning of the new tissue; (ii) adequate porosity and permeability for nutrients and oxygen supply, waste removal, and the release of growth factors; (iii) suitable hydrophilic surface for cell attachment, differentiation, and growth; and (iv) controlled degradation.

Bone is a natural composite consisting mostly of hydroxyapatite (HA), a ceramic based on calcium and phosphate, in addition to proteins and other inorganic compounds. An ideal bone scaffold should aim to mimic this natural composition. Biopolymers, especially thermoplastics, such as polyglycolide (PGA), poly(lactic acid) (PLA), poly(caprolactone) (PCL), and poly(lactic-*co*-glycolic acid) (PLGA), have been commonly utilized in research because of their biocompatibility and their ability to degrade with/over time, providing a temporary artificial medium for the bone healing process until the formation of new bone tissue.^2^

Besides biocompatibility, arguably, one of the most important morphological properties of an ideal scaffold is its porosity, which facilitates the migration and proliferation of mesenchymal cells as well as the formation of vascular and angiogenic networks within tissue.^3^ Consequently, extensive research has focused on scaffold porosity in terms of ideal pore sizes, which range between approximately 100 and 300 μm in diameter for cell migration and delivery of molecules through the scaffold. For processes like vascularization, angiogenesis, and the formation of new bone, pores larger than 300 μm are needed.^4^ However, it is worth noting that even smaller pores ranging between 10 and 75 μm may still lead to the formation of fibrous tissue.^5^ Therefore, to facilitate effective bone healing artificial scaffolds with desired multifunctionality, among other features, must meet pore size requirements encompassing pore sizes in the macro (>100 μm) and micro scales (tens of μm), a concept often referred to as “multiscale” porosity.

Porosity and pore size directly affect a scaffold’s surface characteristics, thereby altering its biological and mechanical functions. For instance, the presence of micropores (<50 μm) gives rise to a larger surface area and may result in increased surface roughness. This, in turn, affects the surface hydrophilicity or wettability typically assessed through changes in the contact angle.^6^ These alterations can lead to an increase in cell attachment, cell proliferation, and cell differentiation.^7^ Indeed, porosity and pore size also play a crucial role in determining a scaffold’s mechanical integrity and strength. There exists an inverse relationship between porosity and strength, signifying that as the porosity increases, the strength of the scaffold tends to decrease. In addition, Shin et al. showed that the mechanical properties of three-dimensionally macrochanneled PCL scaffolds improve with decreasing overall porosity that results from increasing PCL concentration.^8^

As per the review by Bobbert and Zadpoor, for homogeneous, uniform, and efficient cell seeding, pore sizes around 116 μm are preferable; pores smaller than 84 μm and larger than 162 μm lead to inhomogeneous or inefficient cell seeding.^9^ Nevertheless, Murphy et al. discovered that the highest number of cells was found in scaffolds with a mean pore size of 325 μm rather than in the range of 85–190 μm.^10^ While a significant level of porosity is cited to be necessary for even cell distribution within the scaffold,^11^ excessively high porosity (from 71 to 96%) resulted in low seeding efficiency,^9^ possibly due to the decline of the surface area where cells would adhere to. It is crucial for micropores to be interconnected as this is necessary for nutrient diffusion and, consequently, cell viability.^12^ Interconnected micropores enhance cell seeding,^13^ and insufficient interconnectedness results in nonuniform cell spreading.^11^ These morphological parameters together play important roles in tailoring a material’s stiffness and permeability.^13,14^ Therefore, the controlled interconnected porous microstructure stands out as the most important geometrical feature of multifunctional scaffolds to affect the bone regeneration process.

The literature presents a range of techniques aimed at producing porous scaffolds of different scales to improve biological activities and healing in bone tissue engineering. These include methods like polymer impregnation^15^ and hybrid methods such as solvent casting and particulate leaching,^16^ wired network modeling (WNM),^17,18^ and electrospinning^19−21^ as well as various forms of solid freeform fabrication techniques.^22−25^ Among these, some techniques such as gas foaming and salt leaching^26^ and salt leaching using powder (SLUP) and WNM^27^ are one step ahead in that they can be used to form not only macropores but also micropores.

A more recent technique known as nonsolvent-induced phase separation (NIPS) offers distinct advantages compared to other fabrication techniques when it comes to creating micropores in polymeric scaffolds. NIPS is an effective technique that can be used to produce films with micropores, mostly in the form of membranes.^28−30^ Furthermore, it can be seamlessly integrated into the 3D printing process to produce scaffolds with controlled multiscale porosities.^31,32^

Integration of the NIPS technique with 3D printing toward producing scaffolds with controlled macropores alongside micropores was studied by Kim et al.^31^ In this study, PCL-HA scaffolds were produced, demonstrating the ability of the NIPS method to induce micropores into the scaffolds. In another study of the same group, mechanical properties and internal pore structures of PCL scaffolds were tailored by changing the water content in the ethanol-based coagulation bath.^33^ They also fabricated PCL-calcium phosphate (CaP) composite scaffolds with multiscale porosity utilizing camphene as the pore-regulating agent.^34^ However, to the best of our knowledge, no study has examined the effect of the thickness of PCL-HA scaffolds on scales that are appropriate for bone scaffold applications. Also, the literature appears to lack an in-depth analysis and understanding of the internal microstructure morphology of bone scaffolds such as pore orientation, pore size distribution, and anisotropy, which are known to affect the bone regeneration process.

To address this research gap, this study provides a comprehensive investigation of pore morphology and its dependence on experimental parameters in PCL-HA composite scaffolds that were produced via NIPS. More specifically, we explore the effect of nanohydroxyapatite (HA) content and scaffold thickness on the structure of the pores, the pore size distribution, the overall porosity, and the pore anisotropy of these PCL-HA scaffolds.

It is worth noting that in studies investigating the effects of porosity in substrates produced via NIPS, the majority of work has focused predominantly on membranes, with the thicknesses ranging from 70 to 250 μm.^28,35−39^ Very few studies have investigated thicker (i.e., of thickness in the range of mm or more), nonmembranous substrates.^31,40^ Furthermore, most studies have tended to characterize or provide a limited number of cross-sectional views of their substrates using SEM, overlooking the rest of the internal structure. In light of these, this study represents an initial effort to fabricate substrates tailored for bone tissue engineering rather than for thin membranes. Therefore, we refer to these substrates as “thick” films that exhibit microporosity, and this work aimed to delve into their internal pore morphology and how this relates to thickness as well as HA content using primarily micro-CT tomography and SEM. For the sake of clarity, the thicknesses will be referred to as “1 mL” and “2 mL” throughout this paper, corresponding to 1 mL of solution with a thickness of 800–900 μm, and 2 mL of solution with a thickness of 1600–1800 μm, respectively.

## 2 Materials and Methods

### 2.1 Solution Preparation

First, PCL pellets (Mn = 80,000; Sigma-Aldrich, St. Louis, MO, USA) were added slowly into THF solvent in beakers, in 10% (w/v) concentration, and stirred for 24 h at 40 °C. HA powder (<200 nm; Sigma-Aldrich, St. Louis, MO, USA) was added into separate beakers containing the same THF-PCL solution to attain the final HA concentrations of 0, 10, 15, and 20 wt %, and the solutions were stirred for another 24 h at 40 °C. They were then left to cool down. The beakers were sealed tightly with aluminum foil and Parafilm to prevent THF evaporation throughout the solution preparation process.

### 2.2 Scaffold Fabrication

The solutions were transferred into small beakers as 1 or 2 mL using a glass measuring pipet once their temperature dropped to room temperature. Then, the beakers were filled with ethanol. As the ethanol evaporated, the solutions solidified via the NIPS process. When the evaporation completed, the round-shaped samples at the bottom of the beakers, in this paper referred to as “film scaffolds”, were removed from the beakers and left to dry under the hood. HA contents and scaffold thicknesses changed only among the scaffolds, and they remained constant within each scaffold. It is noteworthy that all scaffold combinations, regardless of their thickness and HA content, maintained a 10% polycaprolactone (PCL) concentration in the slurry.

**Figure 1:**
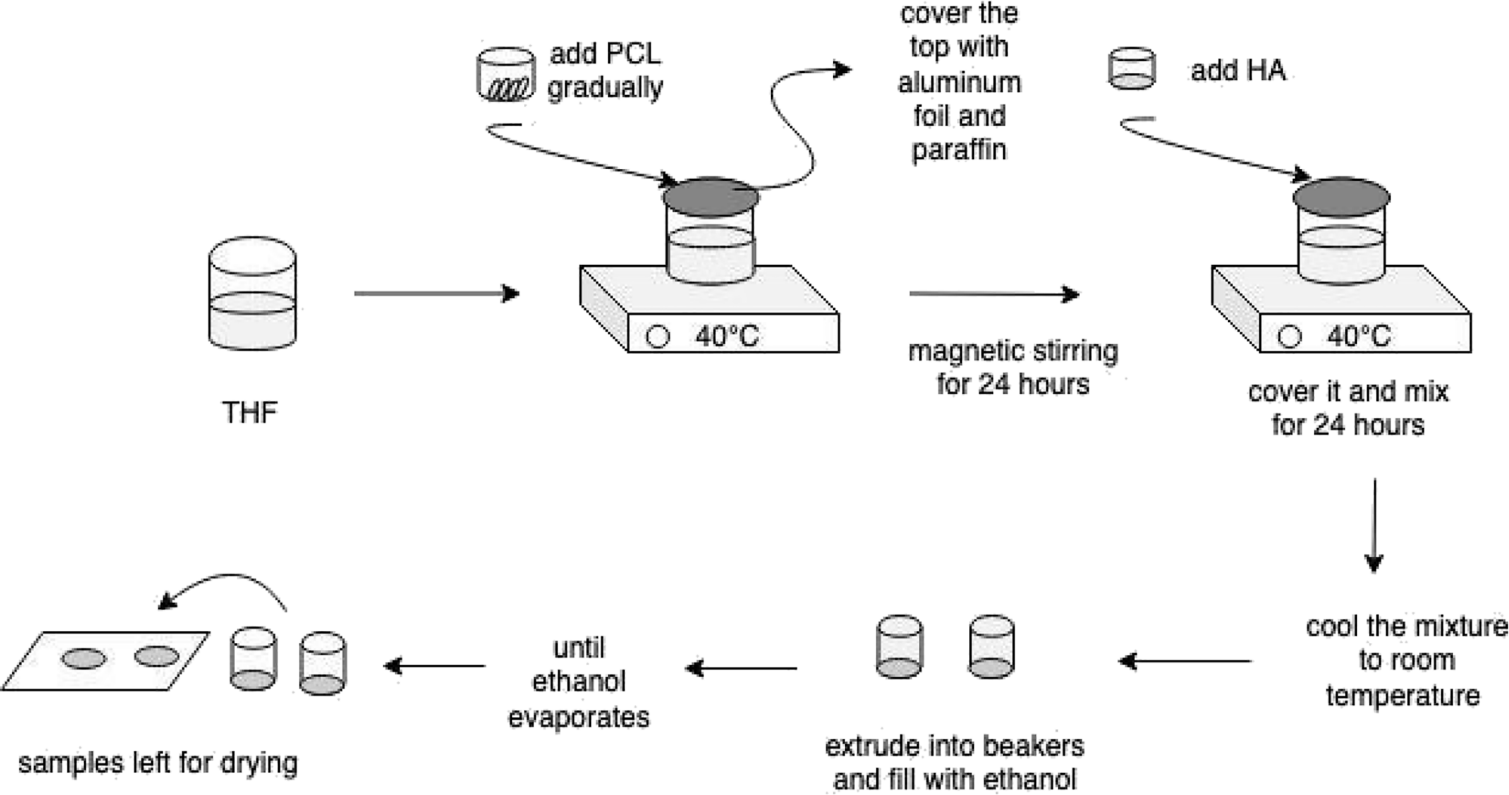
Schematic representation of solution preparation and scaffold fabrication.

A representative scheme of solution preparation and scaffold fabrication is shown in Figure 1.

### 2.3 Characterization

#### 2.3.1 Morphological Characterization with Micro-CT

Micro-CT (SkyScan 1172; Bruker micro-CT, Kontich, Belgium) was used for the morphological characterization of the film scaffolds. More specifically, these scaffolds were characterized and analyzed for their overall porosity, pore size distribution, pore shape, and pore network anisotropy. After the micro-CT scan, reconstruction was performed on the produced 2D slice images using SkyScan NRecon reconstruction software and the 3D internal microstructure of the scaffolds was produced. For inspection purposes, images of the scaffold cross sections were viewed and analyzed by using DataViewer software. Afterward, all quantitative postprocessing was performed using CTAn software on selected 101 slice representative images. Using these 101 slices, the boundaries of a region of interest were defined to create a representative volume of interest (VOI). For further analysis of a binary image format, the original CT image was transformed by setting the lower and upper threshold values to “auto” and 255, respectively. For the anisotropy analysis of the pores in the scaffolds, a two-step procedure was followed: Initially, Otsu’s threshold method of Y. et al. was applied to selected VOIs via the custom processing interface, and the inverse of ROI images was taken. As a result, quantitative 3D analysis was performed to extract all relevant morphological parameters of the pore network, including anisotropy. As a final step, microstructure images of the scaffold’s pore network were obtained by using a 3D modeling tool.

In the micro-CT imaging process, a SkyScan 1172 tomography device was configured with specific scanning parameters. The camera was operated with a pixel size of 8.75 μm, providing detailed resolution at 1336 × 2000 pixels. The resulting image had an effective pixel size of 12.95 μm. To capture optimal data, a source voltage of 90 kV was applied, coupled with an exposure time of 370 ms. The rotation step was set at 0.4°, ensuring comprehensive coverage. Notably, no additional filtration was used during the scan. In the subsequent reconstruction phase, NRecon software was employed, implementing ring artifact correction at a level of 6 and reducing beam hardening by 20%. This led to final images with a resolution of 2000 × 2000 pixels, providing a detailed and accurate representation of the scanned samples.

Another scaffold property known as bone mineral density (BMD) was analyzed by using micro-CT imaging and calibration using available phantoms. BMD refers to the mineral amount in the bone, measured based on the HA amount within the scaffolds. Comparative analysis of HA percentages was presented for the amounts used during solution preparation of the NIPS procedure and the calculated BMD percentage in the scaffolds by using micro-CT tomography. Regarding the latter, BMD calculations were obtained via calibration performed using Bruker micro-CT 1172 and scanning of 0.25 and 0.75 g cm^–3^ HA phantoms. Otsu’s thresholding method was used after the calibration, and the Hounsfield unit and BMD of film scaffolds with various HA contents (0, 10, 15, and 20 wt %) were calculated by CTAn software (version 1.18) for constructed VOIs. BMD was plotted according to the obtained results. Regarding the experimental amount used in the NIPS process, theoretical BMD values were calculated by using standard weight and BMD definitions, as given in eqs 1 and 2:
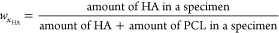
1
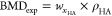
2where *w*_*x*_HA__ is the weight fraction of HA and BMD_exp_ is the bone mineral density of the scaffolds based on their HA density fraction ρ_HA_.

#### 2.3.2 Scanning Electron Microscopy with EDS

Scanning electron microscopy (field emission SEM; Zeiss, Leo Supra VP 35) was used to view the scaffold’s top, bottom, and cross-sectional surfaces and conduct porosity analysis. To perform cross-sectional image analysis, scaffold samples were carefully cut by exposing scaffolds to liquid nitrogen and sputtered by gold–palladium with Denton vacuum sputtering equipment for 135 s. The working distance and the acceleration voltage were set to 12–15 mm and 2 kV, respectively. SEM with EDS analysis was performed to quantify the presence of HA by locating calcium and phosphate and calculate the density of HA.

#### 2.3.3 Mechanical Properties Using the Universal Tensile Test

Zwick/Roell Universal Testing Machine (Ulm, Germany) was used to perform tension tests on the film scaffolds. Uniaxial elongation was applied with a cross-head speed of 2 mm min^–1^ using a 200 N pneumatic load. Tensile stress versus elongation data were recorded during the experiment.

#### 2.3.4 Contact Angle Measurement

To analyze the hydrophilicity of the film scaffolds, a water contact angle measurement was performed using a sessile drop method (Attension, Theta Lite). About 5 μL of distilled water was dropped onto flat film samples. Three measurements were conducted, and the average contact angles of each sample set were recorded.

## 3 Results and Discussion

Here, the results for the fabricated film scaffolds are presented in the following sequence: Initially, a comprehensive morphological analysis of the film scaffolds is presented, including insights into pore network anisotropy, pore shape morphology, and pore distribution, as well as an overview of overall porosity. Our analysis of the effect of thickness and HA content, particularly on pore size distribution, is presented/described. A comparison of the bone mineral densities for scaffolds with varying HA concentrations is provided. This is followed by the presentation of surface and cross-sectional images captured using SEM. Finally, the UTM results, including ultimate tensile strength and Young’s moduli of the scaffolds, are given.

### 3.1 Morphological Analysis of Film Scaffolds with Micro-CT

In this section, we present the micro-CT characterization results obtained for the film scaffolds. More specifically, the results for anisotropy characterization, micro-CT cross-sectional images of the pore morphology, pore size distribution, and overall porosity were presented with an emphasis on the HA content and scaffold thickness effect.

#### 3.1.1 Pore Shape Morphology and Distribution

**Figure 2:**
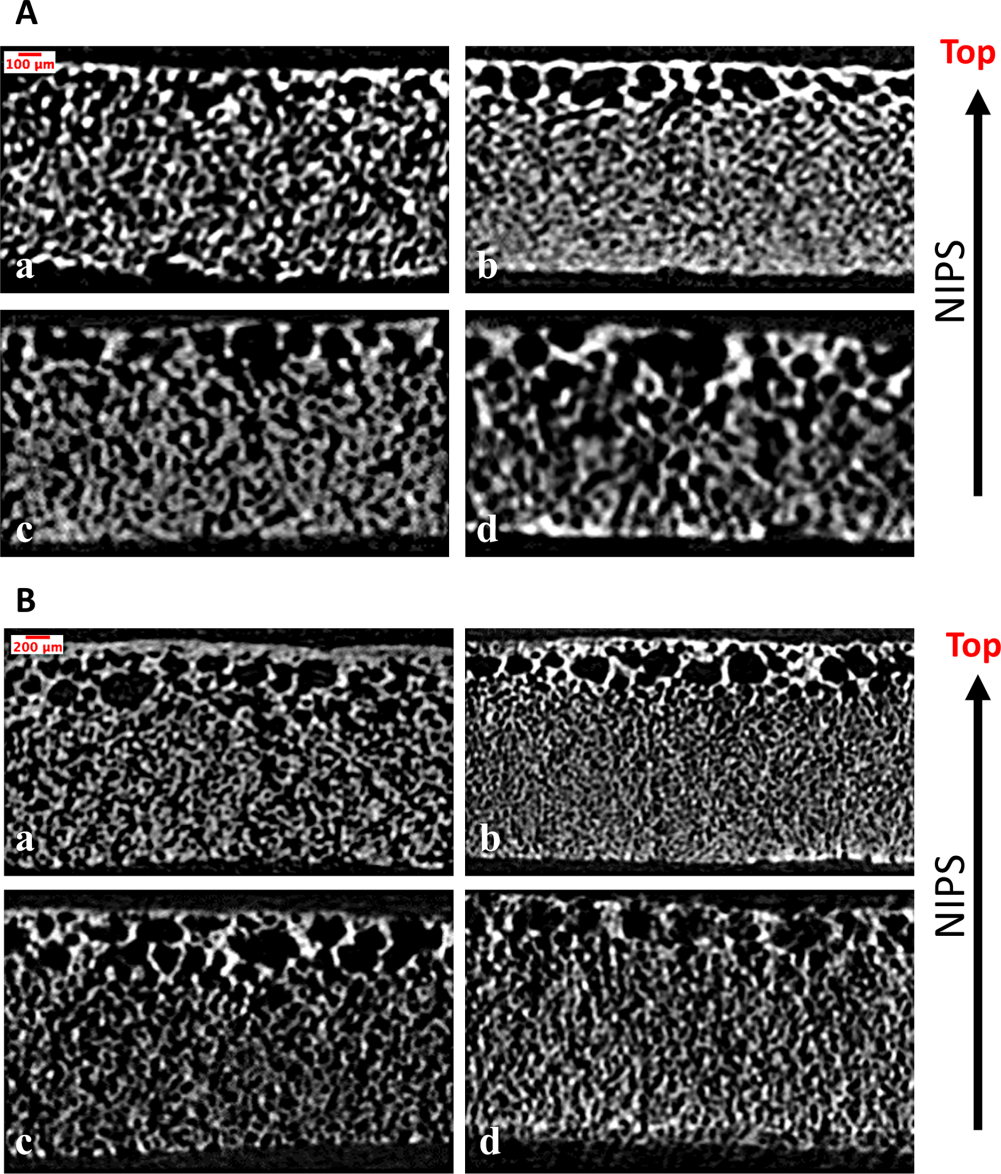
Micro-CT reconstructed images of film scaffolds (A) single (1 mL) and (B) doubled thickness (2 mL) with various HA contents of (a) 0 wt %, (b) 10 wt %, (c) 15 wt %, and (d) 20 wt %.

As regards the pore morphology and distribution, micro-CT reconstructed images shown in Figure 2A,B demonstrated that scaffolds had a higher porosity and a larger pore size near the top/open surface due to the higher interaction and mutual affinity between solvent and nonsolvent. More specifically, the top surface and neighborhood constituted polymer-poor phase characteristics (i.e., the polymer concentration was low), where the phase exchange process was enhanced. The resulting images depicted the film scaffolds with a distinctive finger-like interconnected macro void morphology and a highly porous structure just beneath the top surface. However, the very thin, less porous top surface was attributed to the instantaneous demixing and surface shear. Macro voids appeared and pore size became smaller toward the bottom of the scaffold, which was a polymer-rich phase (i.e., the polymer concentration was high). Micro-CT images also showed that pores were reducing in size when the concentration of HA increased from 0 wt % in one scaffold to 10 wt % in another. On the contrary, their size increased with an increase of HA content above 10 wt %, namely, at 15 and 20 wt % scaffolds. Moreover, a comparative analysis of Figure 2A,B also showed that between the groups of the same amount of HA content, larger pores were induced, and pores were scaled up when the thickness of film scaffolds doubled (from 1 mL thickness to 2 mL thickness).

#### 3.1.2 Effect of Thickness on Pore Size Distribution

**Figure 3:**
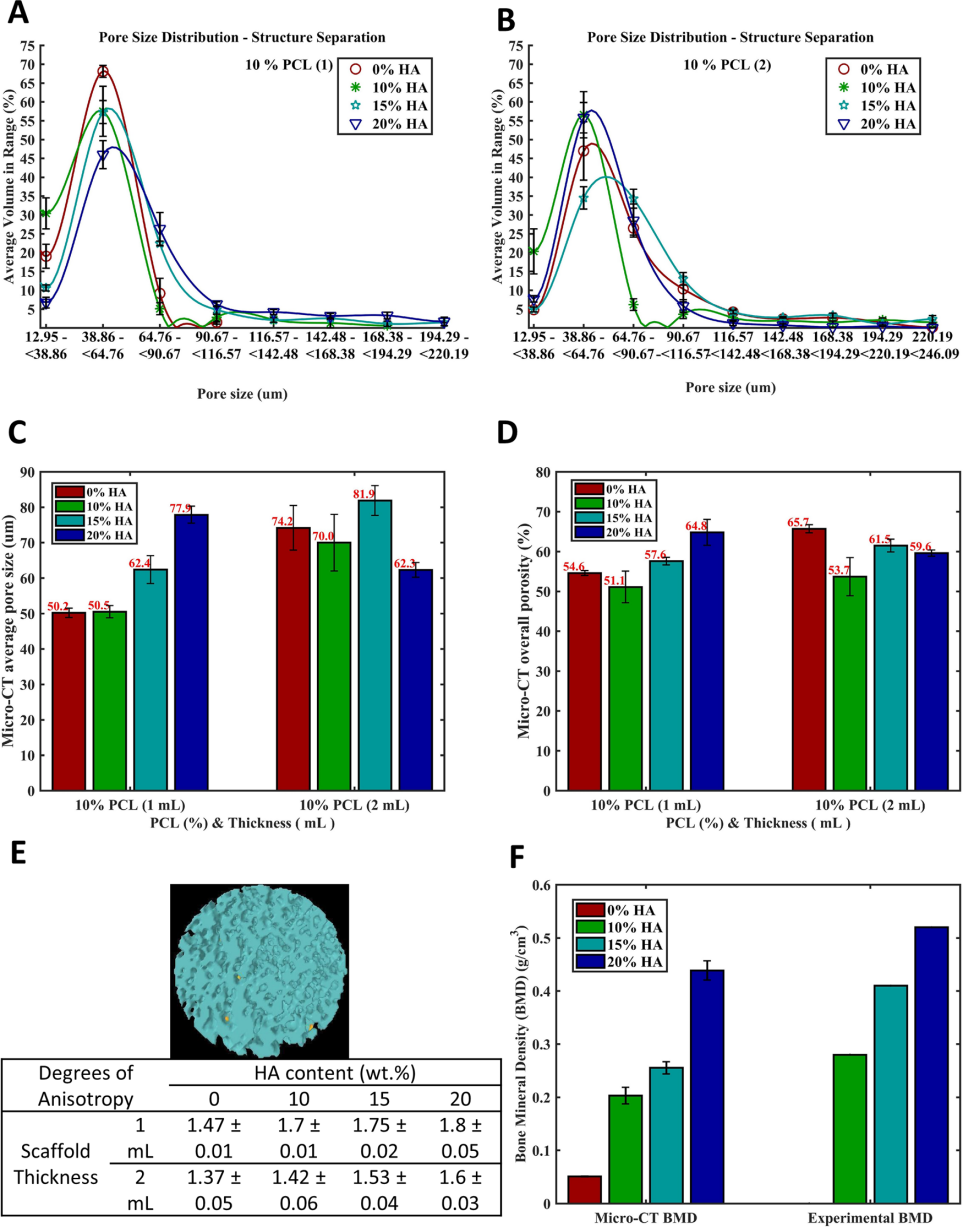
Micro-CT quantitative 3D pore morphology analysis of film scaffolds with various HA contents of 0 wt%, 10 wt%, 15 wt%, and 20 wt%. (A) Micro-CT average pore sizes of film scaffolds for single (1 mL) and doubled (2 mL) thickness. (B) Micro-CT overall porosity of film scaffolds for single (1 mL) and doubled (2 mL) thickness. (C) and (D) Average volumes of pores corresponding to a range of different pore sizes of thinner film scaffolds single (1 mL) and doubled thickness (2 mL), respectively. (E) Representative image and the table of Degrees of Anisotropy of Pore Network. (F) Bone mineral density (BMD) values (via CTAn) and experimental measurement of film scaffolds.

Compared to 1 mL scaffolds, 2 mL scaffolds had larger pores regardless of the concentration of HA (Figure 3A,B). Specifically, scaffolds with 0 and 10 wt % HA did not exhibit pores larger than 116.57 μm for the 1 mL thickness. However, with the increase of thickness by 2-fold, scaffolds exhibited pores larger than 116.57 μm. As can be seen from the comparison of Figure 3A,B, curves slightly shifted from the left (region of smaller pores) to the right (region of larger pores), resulting in a broader pore size distribution. In addition, when the scaffold thickness was doubled, new “largest size” pores were observed with a size larger than 220 μm, as indicated on the edge of the *x*-axis of Figure 3B. The main reason for new larger pores emerging and the overall higher pore size in 2 mL scaffolds might be attributed to the increase of the interaction between solvent and nonsolvent, thereby giving rise to a higher amount of solvent within the deposited solution taking place in the phase separation process.

#### 3.1.3 Effect of HA Content on Pore Size Distribution

Since the effect of scaffold thickness seemed to be dominantly governing the increase of pore size when compared with the effect of HA amount, the isolated effect of HA concentration on the pore size was tested on the single thickness (1 mL) scaffolds shown in Figure 3A. In this case, it was observed that the HA concentration increase from 0 wt % in one scaffold to 10 wt % in another induced the initial average volume in the pore size range (12.95–38.86 μm) to jump from 20 to 30%, denoting a higher number of smaller pores. Also, 10 wt % HA did not improve pore size by forming larger pores compared to those in 0 wt % HA scaffolds most likely because of the too-low concentration of HA not exceeding the threshold. However, when the HA concentration among/of separate scaffolds increased from 0 to 15 wt % and further to 20 wt %, the peak of average volume in the pore size range (38.86–64.76 μm) of scaffolds dropped from 67 to 57 and 47%, respectively. This outcome indicates that the percentage of the smallest pores in the corresponding range decreased as soon as the HA concentration slightly increased and hit the threshold (from 10 to 15 wt % and beyond to 20 wt %) in the scaffolds. The formation of larger pores was depicted by the peak (maximum) spreading to the right-hand side of the graph. In addition, the initial average volume in the pore size range (12.95–38.86 μm) diminished from 20 to 10% and to 5% as the HA concentration increased from 0 to 15 wt % and to 20 wt %, respectively, indicating that the concentration of the smallest pores decreased. Overall, it can be stated that as the HA concentration climbs beyond 10 wt %, pore sizes seem to be enhanced. The same interpretation is valid for the results shown in Figure 3C where the average pore size in micrometers with respect to different HA concentrations and scaffold thicknesses was shown. To sum up, for the effect of HA concentration on pore size, the 1 mL (single thickness) substrates showed that the 10 wt % HA concentration was not enough to make any difference in average pore size (Figure 3C). However, beyond 10 wt % (for 15 and 20 wt % HA scaffolds), the average pore size increased as the HA concentration increased. As mentioned before, in 2 mL thickness substrates, it was the effect of thickness that dominantly led to pore size variations when compared to the effect of the HA concentration.

#### 3.1.4 Overall Porosity

The overall porosity of scaffolds was affected and regulated by the presence and amount of HA content. According to Figure 3D, the scaffolds showed a continuous increase in overall porosity as the HA content escalated from 10 to 15 wt % and to 20 wt %. It is noteworthy that scaffolds having 10 wt % HA content exhibited minimum overall porosity due to the low concentration of HA below the effective HA threshold. However, other than scaffolds with 10 wt % HA content, the overall porosity increased from 54.6 to 57.6% and to 64.8% as the HA concentration increased from 0 to 15 and to 20 wt %, respectively. Scaffolds (2 mL) with a higher HA content had a higher overall porosity as well, except for the highest amount of HA that is 20 wt %. The reason for this behavior may be that thicker scaffolds require higher amounts of solution for deposition and therefore contain higher amounts of solvent in the ethanol bath. This overall increase may result in the enhancement of the interaction between solvent and nonsolvent phases, leading to an enhancement in the phase exchange taking place between them. However, in the case of a higher HA content of 20 wt %, it was observed that the overall porosity decreased with thickness probably due to the presence of higher-than-threshold amounts of HA that resulted in an overall high concentration of solution used for the film scaffold fabrication.

#### 3.1.5 Anisotropy of the Pore Network

A representative image of a film scaffold’s 3D model of the pore network is shown in Figure 3E, and the degree of anisotropy (DA) values evaluated using micro-CT analysis for the produced film scaffolds with 1 and 2 mL thicknesses and varying HA contents are shown in the table in Figure 3E. The corresponding micro-CT reconstructed images of these scaffolds are shown in Figure 2A and Figure 2B for 1 and 2 mL thicknesses, respectively. It is evident from both Figure 2A and Figure 2B that phase separation occurs along the *z*-axis (from the bottom to the top of the specimen), and the anisotropy increases with increasing HA content. Consistent with this observation, Mathieu et al. demonstrated a similar anisotropic behavior in pores generated by the gas foaming technique, with orientation along the foaming direction. Similar to this observation, in the study of Mathieu et al.,^41^ pores produced by the gas foaming technique were oriented along the foaming direction and showed anisotropic behavior. The DA results demonstrated that both neat (pure PCL) and composite (PCL with HA) scaffolds exhibited anisotropy (>1) in pore network morphology. However, the pore network of composite specimens showed higher anisotropic behavior in comparison with the neat specimens, especially for 1 mL film scaffolds. The addition of HA resulted in an overall increase of anisotropy and stimulated pore orientation to be in the direction of phase separation. The degrees of anisotropy of film scaffolds were between 1.37 and 1.8 and agreed with the cited range of 1.1 to 2.38^42,43^ in the literature for the characteristic DA of a typical trabecular bone architecture.

### 3.2 Bone Mineral Density Analysis of Film Scaffolds

The bone mineral density results are plotted in Figure 3F. The HA amounts determined using micro-CT analysis and experimental measurements both followed an increasing trend with relatively similar values. Increasing HA content elevated the difference between the measurements and micro-CT analysis, but it was still within an acceptable range considering uncertainties associated with the submicron particle size of the HA constituent, and the micro-CT scanning resolution limit, as well as experimental uncertainties attributed to VOI selection, thresholding, and phantom size, and calibration setting effects.

As the HA content increased from one scaffold to the other, there was a clear trend of augmentation in both the micro-CT and the experimental values. At 0 wt % HA content, the micro-CT measurement was 0.05 ± 0.005 g/cm^3^. Moving to 10 wt % HA content, this value increased significantly to 0.21 ± 0.016 g/cm^3^. At 15 wt % HA content, the micro-CT value further rose to 0.26 g/cm^3^ (±0.012 g/cm^3^), and at 20 wt % HA content, it reached 0.43 g/cm^3^ (±0.018 g/cm^3^).

The experimental measurements showed a similar progression. At 0 wt % HA content, the value was 0, reflecting the absence of HA. With the introduction of 10 wt % HA content, the experimental measurement rose to 0.28. At 15 wt % HA content, it increased further to 0.405, and at 20 wt % HA content, it reached the highest value of 0.53.

This data suggested a positive correlation between the HA content and the measured values, indicating that as the concentration of HA increased among the scaffolds, so did both the micro-CT and experimental measurements. This trend could be attributed to the increased density and structure provided by the higher HA content, resulting in higher measurement values.

### 3.3 Scanning Electron Microscopy and EDS Analysis of Film Scaffolds

**Figure 4:**
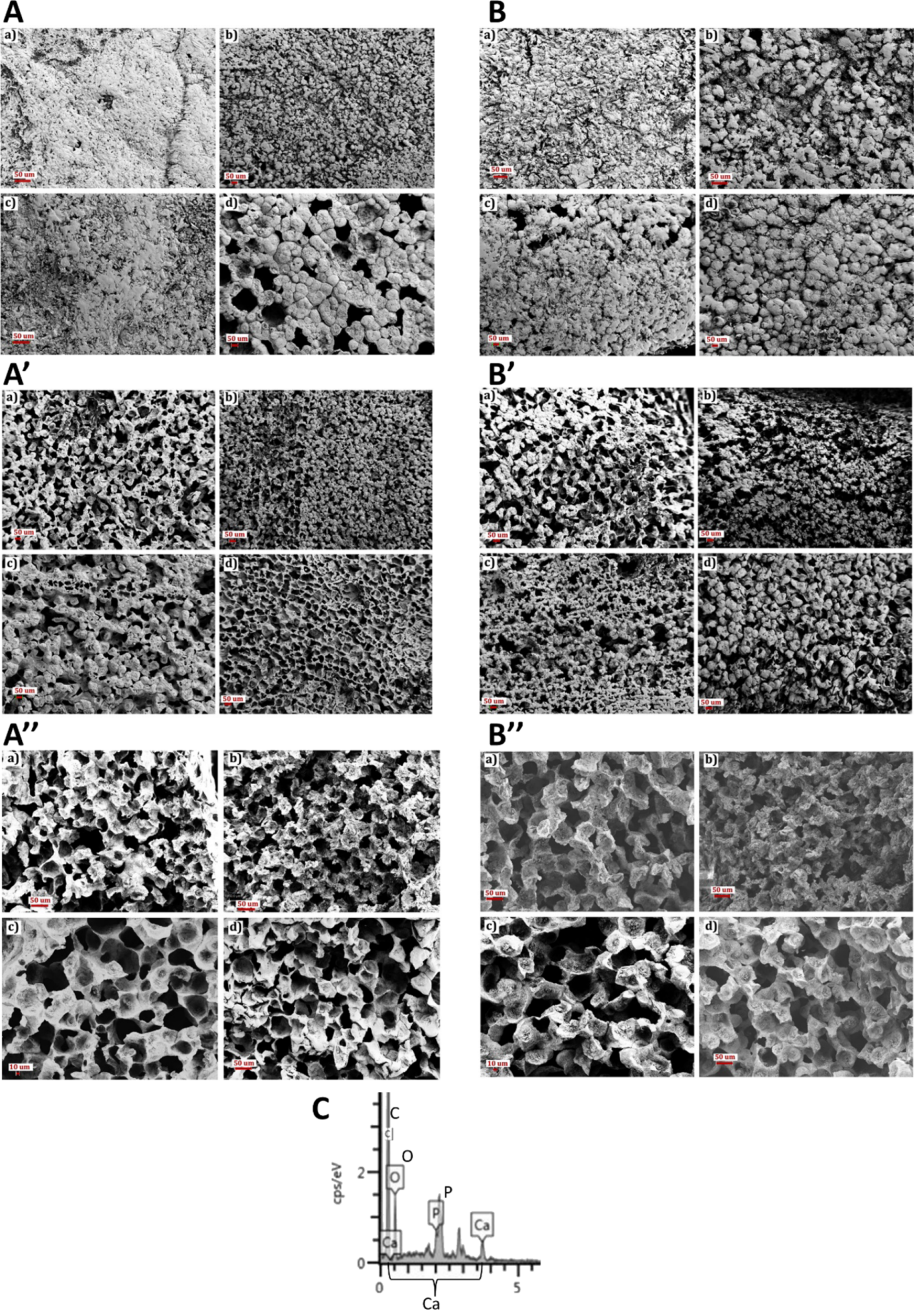
FE-SEM-EDS analysis of film scaffolds with various HA contents of (a) 0 wt %, (b) 10 wt %, (c) 15 wt %, and (d) 20 wt %. The scale bars show 50 μm. (A, A’, A”) Top, bottom, and cross-sectional surface images for the 1 mL scaffold group, respectively. (B, B’, B”) those for 2 mL scaffold groups, respectively. (C) EDS chemical element analysis shows the presence of HA.

The SEM characterization results (Figure 4) demonstrated that micropores were present with a honeycomb-shaped pore morphology and were distributed uniformly throughout the cross section for all film scaffolds with various HA contents (0, 10, 15, and 20 wt %). However, neat scaffolds displayed less top surface porosity than scaffolds containing HA nanoparticles (Figure 4A,B), and the latter displayed a higher pore size on the top surfaces than those of the former. Moreover, images of the bottom surfaces of film scaffolds showed that all specimens exhibited bottom surface porosity regardless of the HA content (Figure 4A’,B). Film scaffolds with 15 and 20 wt % HA content displayed larger pores in diameter at their cross section compared to neat scaffolds and scaffolds with 10 wt % HA content (Figure 4A”,B”). This result is consistent with the structure separation (pore size) distribution results and micro-CT images, as discussed in the previous section. For composition evaluation, EDS analysis was performed. Calcium and phosphate peaks were clearly visible as expected, as shown in the EDS graph (Figure 4C) and BMD plot (Figure 3A).

### 3.4 Mechanical Properties of Film Scaffolds

**Figure 5:**
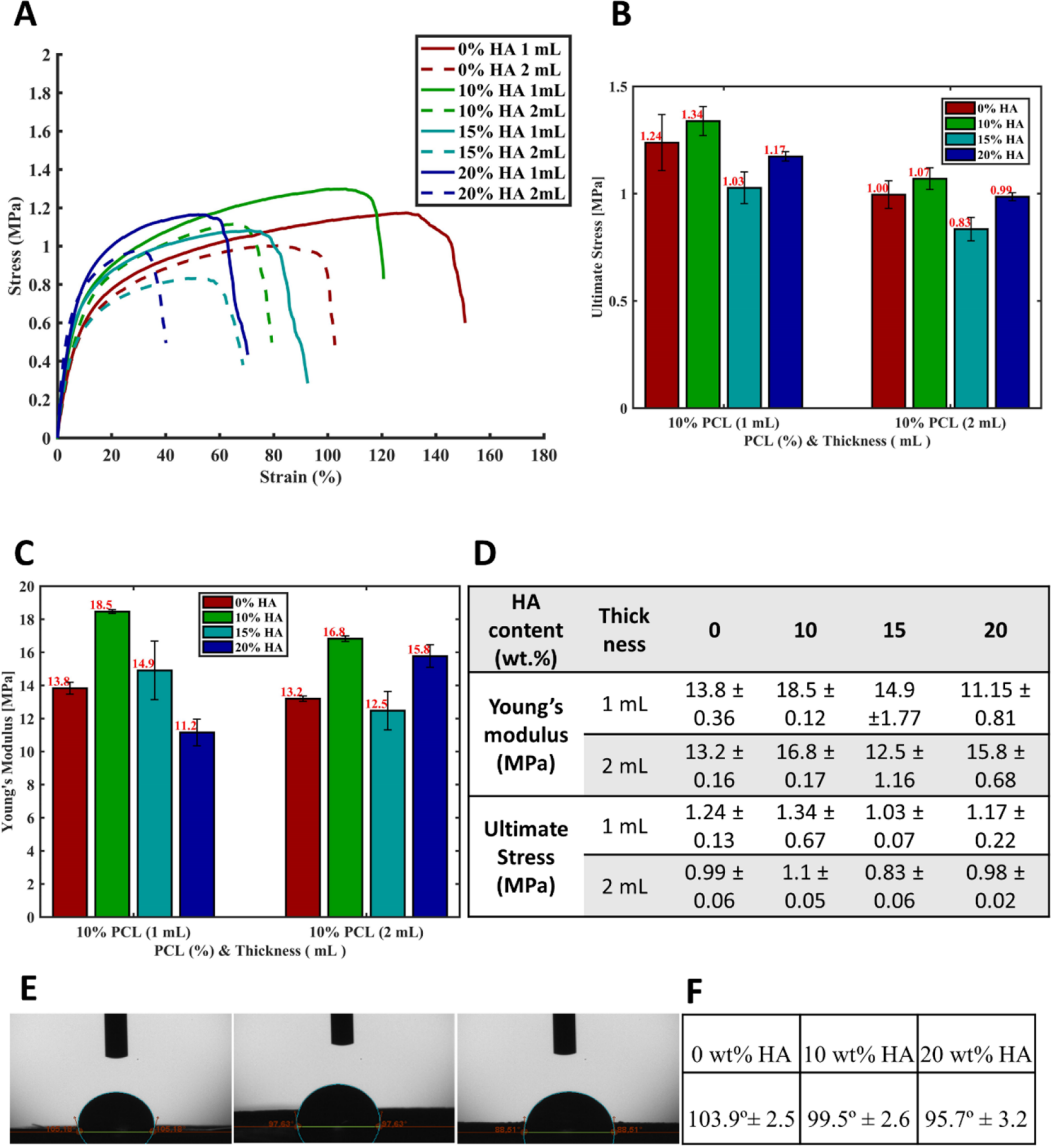
(A) Tensile stress vs strain response of film scaffolds with various HA contents (0, 10, 15, and 20 wt %) for 1 and 2 mL thickness scaffold groups. (B) Ultimate tensile stresses. (C) Tensile Young’s modulus with respect to groups. (D) Summary table for ultimate tensile strength and Young’s modulus. (E, F) Contact angle measurement of film scaffolds with various HA contents (0, 10, and 20 wt %) from left to right, respectively.

Figure 5A depicts a typical stress–strain curve, illustrating how the samples responded to tensile force. Regardless of film thickness and HA concentration, all groups exhibited significant elongation, surpassing a 40% strain value. This behavior indicates the ductile nature of the polymer, resulting in a high toughness, as represented by the area under the curve.

However, the curves also revealed a decrease in the maximum elongational strain with an increase in HA content. This was attributed to the introduction of brittle HA nanoparticles, leading to a loss of ductility in the neat polymer. Likewise, scaffolds containing more HA displayed lower toughness for the same reason. Moreover, an increase in scaffold thickness seemed to adversely affect mechanical properties, as 2 mL scaffolds exhibited larger pores and higher overall porosity compared to those in 1 mL scaffolds, leading to reduced structural integrity.

Among all the HA content and thickness combinations, those with 0% HA and 1 mL thickness exhibited the highest extended strain compared to the others, including its counterpart with a 2 mL thickness. A similar trend of 1 mL substrates (solid lines in the stress–strain curve) showing higher strain/elongation compared to their counterparts with a 2 mL thickness (dashed lines) was observed in all the scaffolds with varying percentages of HA. This phenomenon was attributed to the fact that as the scaffold thickness increased, the mechanical properties of the fabricated scaffolds decreased due to enlarged pores (i.e., larger pore size) and slightly higher overall porosity, which was generally observed in the 2 mL thickness scaffolds, as corroborated by the micro-CT pore calculation results. Consequently, this led to reduced overall structural integrity in the 2 mL scaffolds and a lower capacity for elongation under tensile force.

Adjusting the thickness also influenced the interaction between the solvent (THF) and nonsolvent (ethanol). This is because thickness affects not only the scale of the geometry (and consequently, the scale of induced porosity) but also the depth of penetration in the interaction between the two liquids. This resulted in variations in the pore size and overall porosity throughout the films.

Ultimate tensile strength values and Young’s moduli, as determined by UTM measurements, are presented in Figure 5B–D, respectively. The results indicated that, for scaffolds with a thickness of 1 mL, both ultimate tensile strength and elastic moduli increased from 1.24 ± 0.13 to 1.34 ± 0.67 and from 13.8 ± 0.36 to 18.5 ± 0.12, respectively, as the HA content rose from 0 to 10 wt %. However, beyond this point, Young’s moduli decreased with further increases in HA concentration (Figure 5C,D). Hence, the 10% HA concentration appeared to be the most favorable and optimal choice from a mechanical perspective.

The mechanical performance assessments of scaffolds, detailed in the table in Figure 5, aligned well with the porosity analysis conducted via micro-CT, as presented in the relevant section.

### 3.5 Contact Angle Measurement of Film Scaffolds

Water contact angle measurements were carried out for the film scaffolds as shown in Figure 5E with 1 mL thickness and various HA contents (0, 10, and 20 wt %) and results are shown in Figure 5F. PCL is known as a hydrophobic material, and hydrophilicity was expected to improve with the addition of HA.^44^ Our measurement results shown in the table in Figure 5F demonstrated that as the content of HA increased among the scaffolds, the contact angle and surface hydrophobicity decreased, resulting in an increase in hydrophilicity. These results were in agreement with findings in the literature such as the study by Wang et al.^45^

## 4 Conclusions

In this article, we presented the characterization results of fabricated PCL-HA composite porous bone scaffolds using the NIPS technique. These scaffolds featured varying levels of HA content (0/10/15/20 wt %) and two different thicknesses (1 and 2 mL solutions). Our comprehensive investigation focused on understanding how HA content and scaffold thickness affect the microstructure, surface morphology, and mechanical properties of these scaffolds.

Our results revealed that for thinner substrates, there exists a specific HA threshold that triggers an increase in pore size and porosity. In our study, for 1 mL scaffolds, we observed that pore size increased, and new large pores formed when the HA concentration exceeded or equaled 15 wt %, with further enhancement at 20 wt % HA content. Moreover, increases in scaffold thickness influenced the increase in pore size and distribution to an even greater degree than did the impact of HA content. This effect was especially evident when comparing the results for 2 mL versus 1 mL thick scaffolds.

In line with these observations, below the effective HA threshold value, the UTM results showed that 10 wt % HA scaffolds displayed better mechanical properties than scaffolds with higher concentrations of HA due to the lower porosities and smaller pore sizes of the former. Thus, the mechanical strength of fabricated scaffolds was the highest for 10 wt % HA content, beyond which the addition of HA decreased the ultimate tensile strength and toughness of the tested materials. Similarly, scaffolds with 10 wt % HA content exhibited the highest Young’s moduli. Additionally, micro-CT images demonstrated that all scaffolds had higher porosity and larger pores near the top/open surface due to the higher interaction and mutual affinity between solvent and nonsolvent. For each of the scaffolds, 3D models of the pore network, micro-CT reconstructed images, and DA results showed that pore network orientation was influenced by phase separation, which occurs along the *z*-axis, from the bottom to the top of the specimen. The SEM results demonstrated that the pore morphology consistently resembled honeycomb-shaped macro voids and was uniformly distributed throughout the cross section of all specimens, regardless of their HA content (0/10/15/20 wt %). Additionally, the porosity decreased from the top to the bottom surface of the scaffolds. Contact angle measurements indicated that the hydrophilicity of the scaffolds increased with a higher HA content. This study overall suggests that NIPS is a viable technique for producing microporous composite scaffolds and has the potential to be integrated into 3D printing to produce multiscale porous scaffolds. However, the findings of this study suggest that thickness and HA concentration require careful optimization for desired microstructure morphological characteristics such as porosity, pore size, pore distribution, and mechanical behavior to produce scaffolds using NIPS with desired micromorphological features.
